# Acute effects of a shoe with enhanced plantar sensory feedback on midfoot kinematics whilst walking

**DOI:** 10.1186/1757-1146-4-S1-O2

**Published:** 2011-05-20

**Authors:** Simon Bartold, Adam Bryant, Ross Clark, Kade Paterson, Callan Ritchie

**Affiliations:** 1 The University of Melbourne, Parkville, Victoria, 3010, Australia; 2 Australian Catholic University, Melbourne, Victoria, 3000, Australia

## Background

Excessive foot pronation has been associated with injuries of the lower extremity. No research has investigated the effect of altering plantar sensation on foot pronation. The aim of this study was to determine whether a shoe with enhanced plantar sensory feedback could reduce midfoot pronation.

## Methods

The midfoot kinematics of 21 males were recorded whilst walking in a neutral shoe, a neutral shoe with a foot orthotic and a neutral shoe with nodules located on the plantar medial aspect of the foot (experimental shoe). Electromyography of the peroneus longus, tibialis anterior and medial gastrocnemius was also recorded and analysed. A Friedman’s ANOVA was used to evaluate differences between shoe conditions, and a Wilcoxon signed ranks test was conducted to establish where the differences occurred.

## Results

The results demonstrated that midfoot-tibia angles were significantly more supinated during the loading phase when wearing the experimental shoe (median = 47.70°) than when wearing a neutral shoe (median = 41.50°; *p* = 0.008) or a neutral shoe with a foot orthotic (median = 42.17°; *p*=0.008). In the midstance phase, supination angles in both the orthotic and experimental shoes were higher than that of the neutral shoe, with the former reaching significance (median = 19.56°; *p* = 0.006). During the propulsive phase findings were similar, with a significantly more supinated position in the experimental shoe (median = 48.30) than the neutral shoe (median = 37.47°; *p* = 0.006) or the neutral shoe with a foot orthotic (median = 40.87°; *p*=0.010). No significant differences were observed for any muscle group at any stage of the gait cycle.

## Conclusions

Increasing plantar sensory feedback to the medial aspect of the foot reduces midfoot pronation during an acute bout of walking.this may have important implications in relation to our prescription of both athletic footwear and orthotic devices. Further work is needed to explore whether these effects remain over longer time periods.

